# Expression of ZNF281 in colorectal cancer correlates with response to radiotherapy and survival

**DOI:** 10.1080/07853890.2023.2278619

**Published:** 2023-11-08

**Authors:** Changjiang Qin, Ang Li, Yafei Xiao, Wenjing Liu, Ertao Zhai, Quanying Li, Hong Jing, Yijie Zhang, Hui Zhang, Xuhui Ma, Hongna Tang, Dan Rong

**Affiliations:** a Department of Gastrointestinal Surgery, Huaihe Hospital of Henan University, Kaifeng, China; b Department of Gastrointestinal and Pancreatic Surgery, The First Affiliated Hospital of Sun Yat-sen University, Guangzhou, China; c Department of Pathology, Huaihe Hospital of Henan University, Kaifeng, China

**Keywords:** ZNF281, Colorectal cancer, radioresistance, DNA damage repair, prognosis

## Abstract

**Background:**

The treatment of Colorectal cancer (CRC) is extremely complex and survival rates vary depending on the stage of the disease at the time of diagnosis. Neoadjuvant chemoradiotherapy (NACRT), is the conventional treatment for locally advanced rectal cancer (LARC); however, the resistance to chemoradiotherapy in LARC is difficult to predict.

**Materials and methods:**

In this study, clinical data of 126 LARC patients were collected and analyzed, and relevant validation was performed using GEO database and *in vitro* and *in vivo* experiments, including Western blotting and Real-time quantitative PCR, immunohistochemistry, immunofluorescence, clonogenic cell survival assays, and nude-mouse xenograft models.

**Results:**

In patients with LARC who were treated with neoadjuvant radiotherapy (NART), higher ZNF281 expression in malignant tissue was associated with a poorer prognosis and lesser degree of tumor regression. Cell and mouse experiments have shown that ZNF281 reduces the damage caused by X-rays to CRC cells and tumors grown in mice.

**Conclusion:**

We found that the expression of ZNF281 predicted the radiation response of CRC cells and suggested the prognosis of patients with LARC who received neoadjuvant radiation therapy.

## Introduction

Colorectal cancer (CRC) is the third largest cause of cancer-related mortality globally [1]. Although the prevalence of CRC has decreased in industrialized nations over the last decade, it has risen considerably in China [2]. The National Comprehensive Cancer Network (NCCN) recommendations suggest that neoadjuvant radiotherapy/chemoradiotherapy (NART/CRT) as the primary therapy for patients with locally advanced rectal cancer (LARC) because it reduces the size of the tumor before surgery [3, 4]. The majority of LARC patients treated with NART/CRT demonstrate some degree of tumor response, however some individuals develop resistance to this treatment. Numerous studies have demonstrated that the gene expression pattern of cancer cells influences treatment response in CRC patients, and the use of molecular biomarkers can predict how LARC patients would react to the effectiveness of preoperative irradiation (PRT) [5, 6]. However, limited biomarkers have been verified as radiation response predictors [7].

Zinc finger proteins (ZNFs) belong to a large and diverse family of proteins that possess at least one zinc finger structural domain, which is the largest family of transcription factors in the human genome [8, 9]. Zinc finger proteins perform a wide range of tasks, including RNA packaging, DNA recognition, apoptotic regulation, transcriptional activation, lipid binding, and protein folding and assembly [10]. ZNF281, which is also known as ZBP-99 or ZNP-99, is a 99 kDa zinc-finger transcriptional regulator that binds to a GC-rich region located in the promoter [11]. Several studies have shown that ZNF281 is associated with the regulation of stem cell pluripotency and developmental processes [12–15]. In addition, ZNF281 plays a crucial role in the development and progression of malignant tumors. For example, some studies have reported that ZNF281 can affect the metastasis of HCC and breast cancer [16, 17]. Yu et al. found that ZNF281 promotes the growth and invasion of pancreatic cancer cells through activation of Wnt/β-linked protein signaling [18]. In addition, ZNF281 has been found to be a diagnostic marker for oral squamous cell carcinoma and a prognostic marker for neuroblastoma [19, 20].

Our previous research revealed that ZNF281 is associated with the proliferation, invasion and migration of CRC [21]. In this report, we show that ZNF281 is a radioresistance factor that inhibits radiation-induced DSBs in CRC. In patients who had LARC and were being treated with NART, there was a correlation between a greater level of ZNF281 expression and a weaker tumour shrinkage and a worse prognosis.

## Materials and methods

### Patients

126 LARC patients be diagnosed at Henan University. All patients underwent neoadjuvant radiation (NART) between January 2010 and December 2016 for newly diagnosed LARC. The inclusion criteria were the existence of a single main lesion, the completion of a typical neoadjuvant radiation program, and radical surgical resection. All biopsies were performed prior to NACRT, as stated before [22]. All participants have signed an informed consent form. The Institutional Ethics Review Boards of Henan University authorized the application of tissue pieces (Ethical approval number: 2023223).

### Tumour regression grading

Pathological tumor response to neoadjuvant radiation was determined by post-operative histological exams and grading of 5-grade tumour regression (TRG). The TRG was graded according to the following Dworak scale: Dworak regression Grade 0 (TRG 0), no regression; Grade 1 (TRG 1), tumor mass predominating with fibrosis and/or vasculopathy; Grade 2 (TRG 2), predominant fibrotic alterations with few tumor cells or clusters; Grade 3 (TRG 3), dominant fibrosis outgrowing the tumor mass; Grade 4 (TRG 4): Absence of tumor cells (only fibrotic mass) [23].

### Microarray datasets in GEO database

The Gene Expression Omnibus (GEO) database was accessed in order to get the microarray datasets that were obtained from the human clinical biopsy specimens of rectal tumors. Before patients had preoperative neoadjuvant chemotherapy and radiation treatment, tissue biopsies were performed and specimens were obtained. The relative expression levels of ZNF281 were examined using the GSE133057 dataset [24].

### Immunohistochemistry

IHC assays were carried out as previously reported in detail [21]. First, Slices of tissue that were formalin-fixed, paraffin-embedded, and 4 mm thick were created. Anti-ZNF281 (1:100; ab101318; Abcam), anti-Ki-67 (1:100; #9449; Cell Signaling Technology), or anti-H2AX (1:100; D17A3; Cell Signaling Technology) were all incubated on all slides for an overnight period at 4 °C before being incubated with a secondary antibody (Vectastain ABC kit). Finally, the samples were stained with 3,3-diaminobenzidine (DAB) and counterstained with hematoxylin.

### CRC cell lines

The American Type Culture Collection (ATCC, Manassas, VA, USA) supplied the HCT116 and SW480 human CRC cell lines, which were cultivated in accordance with ATCC guidelines. Both cell lines were shown to be viable by short tandem repeat analysis (China Center for Type Culture Collection, Wuhan, China). The cells were grown in DMEM (DMEM, Biological Industries, USA), with 10% foetal bovine serum (FBS, HyClone, USA) and 1% penicillin/streptomycin added as supplements. At a temperature of 37 °C, all of the cells were grown in an incubator with humidified air that contained 5% carbon dioxide. The steps for constructing cells with stable knockdown of ZNF281 (ShZNF281) were as previously described, and qRT-PCR and Western blotting were used to detect the knockdown efficiency of ZNF281 [21].

### Western blotting and quantitative real-time PCR

Proteins were electrotransferred to PVDF membranes after being separated by 8–10% SDS-PAGE (Millipore, Billerica, MA, USA). After blocking for an hour with 5% non-fat dried milk in Tris-Buffered Saline (TBS) containing 0.5% Tween-20 (TBST), the membranes were incubated for an additional hour with a rabbit or mouse horseradish peroxidase-coupled secondary antibody. Enhanced chemiluminescence was used to measure antibody binding (Millipore, Billerica, MA, USA).

qRT-PCR was carried out as previously described after total RNA was extracted [21, 22]. At least three duplicates of each experiment were carried out.

### Immunofluorescence

Cells are moved into confocal dishes. The samples were then permeabilized with 0.5% Triton X-100, fixed with 4% paraformaldehyde, and blocked with 3% BSA. Anti-H2AX antibodies were added overnight at 4 °C. After that, the sample was exposed to DyLight 488 AffiniPure Goat Anti-Rabbit IgG for 1 h at room temperature in the dark. Next, counterstaining with 4′,6-diamidino-2-phenylindole (DAPI) was done. The quantity of H2AX foci (a marker of DSB) per cell nucleus was evaluated using confocal laser scanning microscopy (Zeiss, Germany). 100 nuclei on average each sample are being examined.

### Clonogenic cell survival assay

The clonogenic survival test was conducted as previously reported [22]. Briefly, two hundred transfected cells were seeded in six-well plates. The cells were kept in an incubator at 37 °C with 5% CO2 for 10 to 14 days following the addition of DMSO for 6 h, a variable dose of IR was delivered (0-8 Gy). Finally, after fixation with methanol and staining with crystal violet, colonies with more than 50 cells were counted.

### Nude mouse xenograft model and radiotherapy

Cells were injected subcutaneously into nude mice to generate xenograft models. The mice were treated with X-rays at 5 times the dose of 2 Gy every other day when the tumour volume reached 100 mm^3^. The average tumour volume was measured at each time point. All mice were sacrificed on the 14th day, and the tumours were removed for pathological examination.

### Statistical analysis

Statistical analysis and graphing were performed by GraphPad Prism or SPSS 20.0 software. T test was used to compare two experimental groups. The χ^2^ test was used to assess differences in clinical features. Kaplan-Meier, as well as univariate and multivariate Cox regression, were used for the survival analysis. A value of *p* < 0.05 was considered statistically significant. Similarly, items with *p* < 0.05 in the univariate Cox regression analysis were able to enter multivariate Cox regression.

## Results

### High ZNF281 levels are associated with radioresistance and poor prognosis in patients with LARC treated with NART

**Figure 1.**
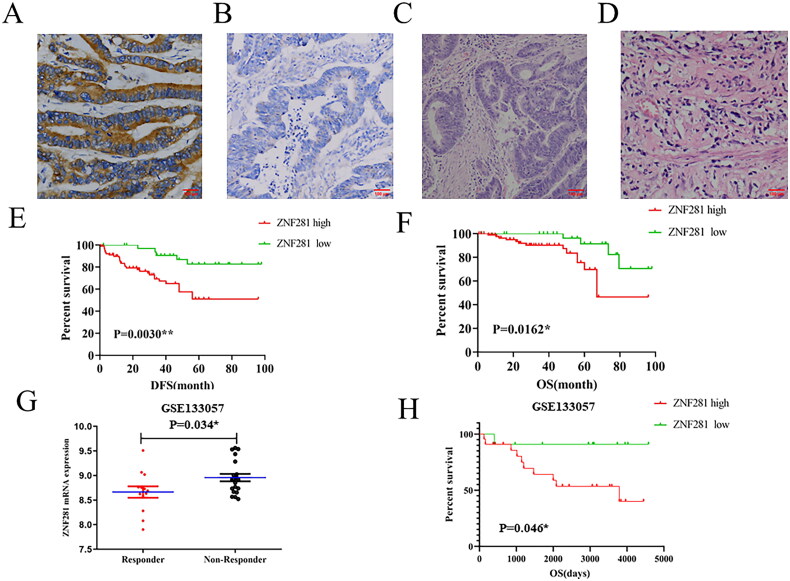
ZNF281 promotes CRC cell radioresistance. (A) Positive ZNF281 expression in a representative biopsy specimen from a patient who exhibited resistance to PRT (×200). (B) Negative ZNF281 expression in a representative biopsy specimen from a patient who exhibited sensitivity to PRT (×200). (C) Positive ZNF281 expression in a representative biopsy specimen with poor tumor regression grade (TRG ≤ 2) (×200). (D) Negative ZNF281 expression in a representative biopsy specimen with good tumor regression grade (TRG ≥ 3) (×200). (E) Kaplan–Meier curves of DFS in our cohort. (F) Kaplan–Meier curves of OS in our cohort. (G) ZNF281 levels in different subgroups (responder, respond to radiation; nonresponder, no response to radiation). (H) Kaplan–Meier curves of OS in GSE133057 dataset. (*p < 0.05; **p < 0.01).

Differentially expressed genes between NART responders and nonresponders may play a crucial role in radioresistance [25]. We analysed a microarray dataset (GSE133057) from the NCBI-GEO database and found significant upregulation of ZNF281 in nonresponders compared to responders (*p* = 0.034) (Figure 1G). This finding suggested that upregulation of ZNF281 might be related to the differential radiotherapy response to NART among rectal cancer patients. According to the analysis of survival, high expression of ZNF281 was shown to be related with a poor outcome in CRC patients undergoing NART (Figure 1G). The high expression of ZNF281 may predict poor tumour response and prognosis in rectal cancer patients receiving NART, encouraging us to explore further the status of the expression and clinical relevance of ZNF281 in rectal carcinoma.

Thus, we analyzed the relationship between ZNF281 expression and tumor regression grade (TRG) in pretreatment biopsies of 126 patients who did receive NART after surgery. Of these patients, 91/126 (72.2%) exhibited positive ZNF281 expression (Figure 1A), and 35/126 (27.8%) patients demonstrated negative expression of ZNF281 (Figure 1B). After PRT, 92/126 (73.0%) instances had an inadequate response (TRG ≤ 2) (Figure 1C), whereas in 34/126 (27.0%) instances there was a favorable pathologic response (TRG ≥ 3) (Figure 1D).

Chi-square test revealed that a high level of ZNF281 was substantially related with TRG (*p* = 0.001), LN metastasis (*p* = 0.046), TNM stage (*p* = 0.036) (Table 1). The Kaplan–Meier logistic regression revealed that individuals with elevated levels of ZNF281 had a shorter OS (overall survival) (Figure 1E and F).

**Table 1.** Chi-square test of clinicopathologic characteristics and ZNF281 mRNA expression in our cohort.

Characteristics		ZNF281 expression			
		High (*n* = 91)	Low (*n* = 35)	χ2	*P* value
**Age**	**<60**	24	9	0.006	0.940
	**>60**	67	26		
**Gender**	**Female**	26	14	1.524	0.217
	**Male**	65	21		
**T stage after pCRT**	**T0-2**	44	18	0.096	0.757
	**T3-4**	47	17		
**N stage after pCRT**	**N0**	62	30	**3.966**	**0.046**
	**N1-2**	29	5		
**TNM stage after pCRT**	**0-II**	61	30	**4.397**	**0.036**
	**III**	30	5		
**Tumor downstage after pCRT**	**0-1**	62	23	0.067	0.795
	**2-3**	29	12		
**Tumor regression grade after pCRT**	**0-2**	74	18	**11.462**	**0.001**
	**3-4**	17	17		

pCRT: preoperative chemoradiotherapy.

By Cox regression, univariate Cox regression analysis revealed that ZNF281 expression level, T stage after pCRT, N stage after pCRT, TNM stage and Tumor downstage after pCRT correlated with OS. Five clinical factors were significant in the results of the univariate COX analysis, which were then included in the multivariate COX analysis. The results of multivariate Cox regression analysis indicated that ZNF281 might be an independent prognostic factor for CRC patients (hazard ratio (HR), 11.419; 95% confidence interval (CI), 1.694-76.961; *p* = 0.012; Table 2). Likewise, TNM stage after pCRT also might be an independent prognostic factor for CRC patients (HR, 9.679; 95% CI, 1.041-89.971; *p* = 0.046).

**Table 2.** Univariate and multivariate Cox regression analysis of clinical pathologic features according to our cohort.

Parameters	OS			
	Univariate		Multivariate	
	HR (95% CI)	*P*	HR (95% CI)	*P*
**ZNF281**	5.494 (1.574-19.173)	**0.008**	11.419 (1.694-76.961)	**0.012**
**(high vs low)**				
**Age**	0.484 (0.186-1.258)	0.136	–	–
**(<60 vs > 60)**				
**Gender**	1.880 (0.663-5.333)	0.236	–	–
**(female vs male)**				
**T stage after pCRT**	6.980 (2.000-24.364)	**0.002**	3.422 (0.696-16.826)	0.130
**(T0-2 vs T3-4)**				
**N stage after pCRT**	3.374 (1.304-8.733)	**0.012**	0.139 (0.015-1.297)	0.083
**(N0 vs N1-2)**				
**TNM stage after pCRT**	4.190 (1.623-10.816)	**0.003**	9.679 (1.041-89.971)	**0.046**
**(0- II vs III)**				
**Tumor downstage after pCRT**	0.093 (0.012-0.697)	**0.021**	0.163 (0.011-2.320)	0.181
**(0-I vs II-III)**				
**Tumor regression grade after pCRT**	0.020 (0.000-1.086)	0.055	–	–
**(0-2 vs 3-4)**				

HR: hazard ratio; CI: confidence interval; pCRT: preoperative chemoradiotherapy.

### ZNF281 knockdown increases radiosensitivity of CRC cells

**Figure 2.**
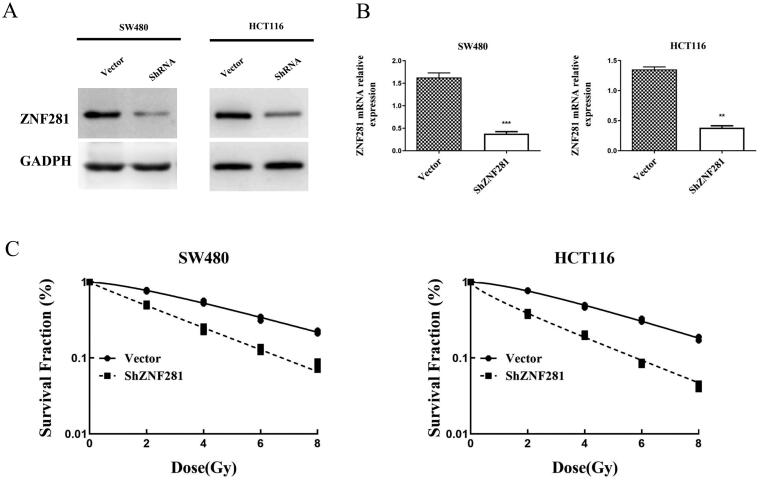
ZNF281 was identified as an anti-radiation factor that improved the survival of CRC cells irradiated by X-rays. (A) Verification of ShRNA efficiency for ZNF281 knockdown by Western blot analysis. (B) Verification of ShRNA efficiency for ZNF281 knockdown by RT-PCR. (C) Colony formation assay was used to assess the survival of CRC cells after 8 days of exposure to X-rays (Vector, lentiviral vector; ShZNF281, ZNF281 after stable knockdown).

Our research identified that ZNF281 is highly expressed in SW480 and HCT116 cell lines. To verify the hypothesis that ZNF281 downregulation sensitizes CRC cells to radiation, we utilized ShRNA to knockdown ZNF281 expression in SW480 and HCT116 cells. Western blot and qPCR analyses confirmed ZNF281 knockdown (Figure 2A and B). Following exposure to different levels of radiation, ZNF281-depleted cells formed considerably fewer colonies, indicating that ZNF281 is essential for the cellular radiation response (Figure 2C).

### ZNF281 facilitates DSB repair in CRC cells

**Figure 3.**
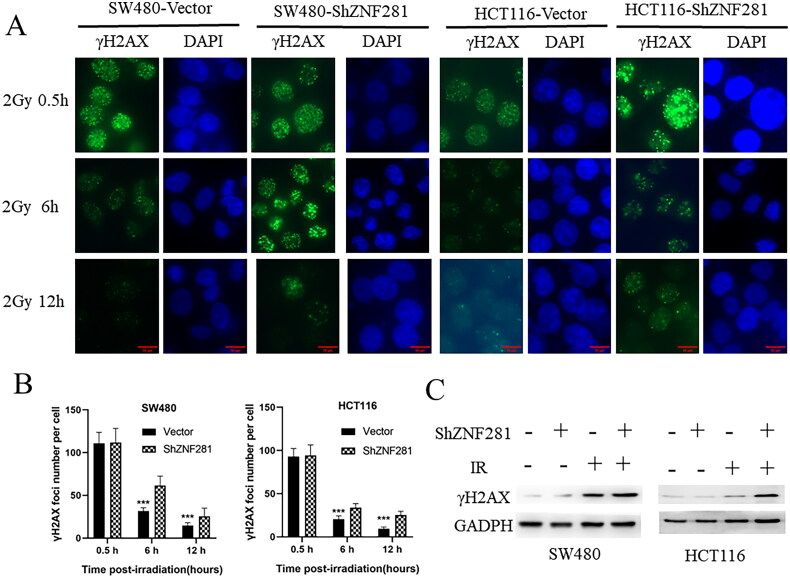
ZNF281 promotes radiation resistance by promoting DSB repair in CRC cells. (A) Immunofluorescence images of γH2AX levels and time course of lesion changes in X-ray-treated CRC cells (×400). (B) The foci number in CRC cells (mean ± SD). (C) The effects of ZNF281 on γH2AX levels in CRC cells treated with X-rays (loading control: GAPDH. Vector, lentiviral vector; ShZNF281, ZNF281 after stable knockdown; ***, *p* < 0.01).

To clarify whether ZNF281 inhibited DNA damage or repair caused by radiotherapy, we assayed γH2AX levels at different times after radiotherapy. The persistence of γ-H2AX foci following IR reflects an impaired cellular capacity to repair DNA DSBs [26–28]. The longer the presence of γ-H2AX, the weaker the repair ability, and the faster the disappearance of γ-H2AX, the stronger the repair ability. After IR treatment, the γ-H2AX level is initially very high and gradually decreases with DNA repair. Compared to control cells, γH2AX levels were reduced more slowly in ZNF281-deficient colorectal cancer cells (Figure 3A and B). Moreover, it’s observed that the level of γH2AX was apparently higher in ZNF281-deficient cells than in carrier cells treated with X-rays (Figure 3C). In summary, these results support that ZNF281 promotes DSB repair.

### ZNF281 affects the response of CRC cells to radiotherapy in a nude mouse model

**Figure 4.**
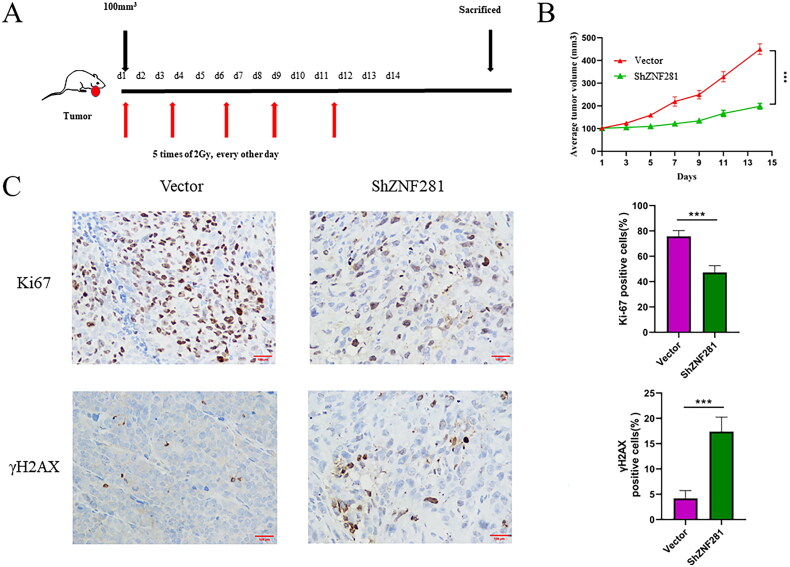
ZNF281 promotes the resistance of CRC cells in a nude mouse model (A) HCT116 cells transfected with ZNF281 low expression (ShZNF281) and vector (vector) to establish transplanted xenografts were treated with X-rays (2 Gy/day) for 14 days. (B) Tumour volumes measured at different time points. (C) Images of IHC staining of Ki67 and γH2AX in xenografts (×200). (***, *p* < 0.001; vector, lentiviral vector; ShZNF281, ZNF281 after stable knockdown).

Next, we evaluated the impact of ZNF281 on radiation response in a nude mouse xenograft model. Mice were treated with X-rays after the establishment of a CRC xenograft (2 Gy/day every other day for a total of five times). Xenografts derived from ShZNF281 cells after radiotherapy were smaller than those derived from carrier cells (Figure 4A and B). Next, we examined the expression of γH2AX and Ki-67 through IHC staining of tumour tissue. The results showed that Ki-67 staining in ZNF281 knockdown xenografts was weaker than that in the vector group, and the opposite effects were found for γH2AX staining (Figure 4C). This suggests that reducing the expression of ZNF281 can effectively increase DNA double-strand breaks and reduce cell proliferation activity after radiotherapy.

## Discussion

ZNF281, an oncogene, is associated with poor prognosis in pancreatic cancer [18], oral squamous cell carcinoma [19], and neuroblastoma [20]. A recent study showed that inhibiting ZNF281 leads to potent radiosensitization of non-small cell lung cancer [29]. Currently, there is a lack of studies on the response of ZNF281 to radiation therapy in CRC. In this study, we determined the antiradiation effect and prognostic potential of ZNF281 in CRC.

PRT has become the standard of treatment for LARC; however, not all patients are sensitive to radiation [30]. TRG is often used to assess the early efficacy of LARC after PRT treatment [31]. Our clinical investigation showed that low expression of ZNF281 was significantly associated with higher TRG scores after PRT, suggesting that patients with low expression of ZNF281 had better early outcomes with PRT treatment (Table 1). In addition, it’s found that high ZNF281 expression was associated with poor OS and DFS in CRC patients who received neoadjuvant radiotherapy (Figure 1E and F). We obtained mRNA data from patients receiving radiotherapy in the GEO database for validation and found that CRC patients with lower ZNF281 expression had better survival outcomes, which was consistent with our results (Figure 1G and H). These results suggest that ZNF281 has the potential to predict whether patients with CRC are radiosensitive or radioresistant.

Radiation causes cellular DSBs, and γH2AX foci and γH2AX levels are sensitive DSB markers [28]. The persistence of γH2AX foci marks a delay in repair and is associated with radiosensitivity [32, 33]. The lack of clearance of γH2AX foci in ZNF281 knockdown cells suggests that HRR is inefficient in them. In a further investigation, we found that ZNF281 knockdown enhanced the radiosensitivity of CRC cells. Moreover, similar to the *in vitro* results, ZNF281 facilitates resistance to X-rays in CRC cells in a mouse model.

RT primarily eliminates cancer cells by producing DNA damage, and double-strand breaks (DSBs) are the most lethal form of DNA damage [34]. DSBs are repaired through two major pathways: nonhomologous end-joining (NHEJ) and homologous recombination repair (HRR) [35].The homologous recombination (HR) repair is one of the principal DSB repair pathways in eukaryotes, and excessive DSBs beyond the ability for repairing would result in apoptosis. Tumor cells with high DNA repair capacity exhibit inherent drug resistance that reduces the effectiveness of this RT, and the reliance on DNA repair pathways makes them a hopeful target for cancer therapy [36–39]. Our previous studies have shown that XRCC2 functions as a resistance factor that protects CRC cells from radiation-induced DSBs and that XRCC2 levels may act as a valid predictive biomarker of radiosensitivity in LARC patients [22]. Recently, Pieraccioli et al. found that ZNF281 promotes DNA damage response by controlling the expression of XRCC2 [40]. Therefore, we speculate that the antiradiation effect of ZNF281 here in CRC may also be related to XRCC2, and we will verify this possibility in the future.

## Conclusion

In summary, ZNF281 acts as a resistance factor against radiation-induced DNA damage *in vitro* and in nude mouse CRC xenografts. Upregulation of ZNF281 in CRC was associated with poor prognosis in LARC patients treated with radiotherapy. Therefore, ZNF281 may serve as a valid predictive biomarker of radiosensitivity and a target for radiosensitization in patients with LARC. These findings may help in the development of precise treatment strategies in future clinical practice.

## Data Availability

The data used to support the findings of this study are included within the article.
